# Referral bias in ALS epidemiological studies

**DOI:** 10.1371/journal.pone.0195821

**Published:** 2018-04-16

**Authors:** Giancarlo Logroscino, Benoit Marin, Marco Piccininni, Simona Arcuti, Adriano Chiò, Orla Hardiman, James Rooney, Stefano Zoccolella, Philippe Couratier, Pierre-Marie Preux, Ettore Beghi, for EURALS

**Affiliations:** 1 Department of Basic Medical Sciences, Neuroscience and Sense Organs, University of Bari “Aldo Moro”, Bari, Italy; 2 Unit of Neurodegenerative Diseases, Department of Clinical Research in Neurology, University of Bari “Aldo Moro”, at “Pia Fondazione Cardinale G. Panico“, Tricase, Lecce, Italy; 3 INSERM UMR1094, Tropical Neuroepidemiology, Limoges, France; 4 University of Limoges, School of Medicine, Institute of Neuroepidemiology and Tropical Neurology, CNRS FR 3503 GEIST, Limoges, France; 5 CHU Limoges, Centre d’Epidémiologie de Biostatistique et de Méthodologie de la Recherche, Limoges, France; 6 Laboratorio di Malattie Neurologiche, Dipartimento di Neuroscienze, IRCCS Istituto di Ricerche Farmacologiche Mario Negri, Milano, Italy; 7 ALS Center, Department of Neuroscience, University of Turin, Turin, Italy; 8 Neuroscience Institute of Turin (NIT), Turin, Italy; 9 Institute of Cognitive Sciences and Technologies, C.N.R., Rome, Italy; 10 Academic Unit of Neurology Trinity Biomedical Sciences Institute Trinity College Dublin, Dublin, Ireland; 11 CHU Limoges, Service de Neurologie, Centre expert SLA, Limoges, France; University of Toronto, CANADA

## Abstract

**Background:**

Despite concerns about the representativeness of patients from ALS tertiary centers as compared to the ALS general population, the extent of referral bias in clinical studies remains largely unknown. Using data from EURALS consortium we aimed to assess nature, extent and impact of referral bias.

**Methods:**

Four European ALS population-based registries located in Ireland, Piedmont, Puglia, Italy, and Limousin, France, covering 50 million person-years, participated. Demographic and clinic characteristics of ALS patients diagnosed in tertiary referral centers were contrasted with the whole ALS populations enrolled in registries in the same geographical areas.

**Results:**

Patients referred to ALS centers were younger (with difference ranging from 1.1 years to 2.4 years), less likely to present a bulbar onset, with a higher proportion of familial antecedents and a longer survival (ranging from 11% to 15%) when compared to the entire ALS population in the same geographic area.

**Conclusions:**

A trend for referral bias is present in cohorts drawn from ALS referral centers. The magnitude of the possible referral bias in a particular tertiary center can be estimated through a comparison with ALS patients drawn from registry in the same geographic area. Studies based on clinical cohorts should be cautiously interpreted. The presence of a registry in the same area may improve the complete ascertainment in the referral center.

## Introduction

Data collected in amyotrophic lateral sclerosis (ALS) tertiary centers are used for descriptive and analytic studies[1, 2], clinical investigations [3] including the construction of clinical practice guidelines [4, 5], randomized clinical trials (RCTs) and other scientific fields like medico-economics [6]. Nevertheless, the investigation of this rare neurodegenerative disease (crude incidence estimated at 2.16/100 000 person-years in Europe [7]) may be reliably quantified only by population-based approach.

Tertiary-based cohort studies have been described as not representative of the true ALS population due to incomplete case ascertainment related to a selection bias known as referral bias [8]. Selection bias is a systematic error in the recruitment process determined by sampling, non-response or loss to follow-up. Referral bias is a specific type of selection bias that determines a real sample different from the target study population in key variables [9].

Referral may also bias the inception of subjects in RCTs, as most clinical studies enrol patients exclusively from ALS tertiary centers. Despite these concerns, the extent of referral bias in studies remains largely unknown.

Several epidemiological registers have been established in Europe to accurately quantify the incidence and clinical features of ALS in the general population using multiple sources of information to ensure complete case ascertainment [10–18]. Using data from four European ALS registry members of the EURALS consortium (pan-European ALS Register) we aimed (i) to measure the extent of referral bias by comparing clinical, demographic features and survival of population-based cohorts with ALS referral cohorts from the same population, (ii) to investigate variation in the extent of bias depending on settings and time.

## Methods

### Settings

Data for this study were obtained from four European ALS registries located in Piedmont (PARALS), in Northern Italy, covering 4 225 023 inhabitants in 2 000 [11]; Puglia (SLAP), in Southern Italy, covering 4,034,132 inhabitants in 2 000 [13]; Republic of Ireland (Irish ALS register), covering 3 626 087 inhabitants in 1996 [10]; Limousin (FRALim), center of France, covering 741 083 inhabitants in 2011 [14].

Time periods covered in Italian or Irish registers (prior to the year 2 000), were different from the Limousin register (after the year 2000). This allowed us to examine possible evolution over time of ALS demographic characteristics and the possible impact on referral.

In the study period, the total amount of person-years considered here is around 50 million.

### Inclusion criteria

Patients were included (i) if they originated from the geographically defined catchment populations at the time of diagnosis of ALS according to (ii) the original El Escorial criteria (EEDC) [19] (i.e. definite, probable, possible or suspected ALS) for PARALS, SLAP, and Irish ALS register, or according to the revised EEDC [20] (i.e. definite, probable, probable laboratory-supported or possible ALS) for FRALim register, and then (iii) if they were identified by at least one of the multiple sources used for case ascertainment.

### Sources of case ascertainment

Multiple sources of ascertainment of cases are described in Table 1. In all cases, registers relied on recruitment by ALS tertiary referral center, neurologists and neurophysiologists, both private and from the national health system, in the geographic area of interest. Hospital discharge data were considered by three registers, while local charitable associations, general practitioners or other specialists in the field were used in some cases.

**Table 1:** Characteristics of European registers participating in the study.

	Piedmont (Italy)	Puglia (Italy)	Republic of Ireland	Limousin (France)
	PARALS	SLAP	Irish ALS register	FRALim
Period of recruitment (years)	1996–1999	1998–1999	1996–1999	2000–2013
Population*	4,225,023	4,034,132	3,626,087	741,082
Person-years	16,900,092	8,068,264	14,504,348	10,004,607
Geographical coverage (km^2^)	25,400	19,345	70,280	19,942
Sources of case ascertainment				
ALS tertiary referral center	X	X	X	X
Neurologists	X	X	X	X
Other specialists in the field (neurophysiologists, neurosurgeons, pathologists)			X	
Primary care physician			X	
Hospital discharge data	X	X		X
Heath insurance				X
Local charitable Organization/ALS association		X	X	

* Population for Ireland in 1996 (http://www.cso.ie/en/census/census1996reports/census1996principaldemographicresults/), Population for Piedmont and Puglia in 2000 (http://demo.istat.it/ric/index_e.html), Population for Limousin in 2011 (http://www.insee.fr/fr/themes/detail.asp?ref_id=estim-pop&reg_id=99)

### Description of data available for each patient

The following information was available for each patient: age at diagnosis (years), family history of ALS (fALS), sex, site of symptom onset (defined as bulbar-onset, limb-onset, generalized, respiratory-onset),diagnostic delay (from date of symptom onset to date of diagnosis, months), EEDC or EEDC-revised classification at time of diagnosis, date of death or last known to be alive. Date of diagnosis represented the date in which the patient was informed of his/her diagnosis.

### Definitions

In this study, we compare the baseline demographic and clinical characteristics along with outcomes (i) of patients diagnosed and followed by the tertiary referral center in the geographical areas considered, with those (ii) of the entire ALS population recruited by the local registers using multiple sources. Patients that make up the tertiary referral cohort were also part of the population-based cohort for that region.

Within each region, there is a referral multidisciplinary tertiary center, which take care exclusively to ALS patients. A patient was defined as an attendee at the tertiary referral center if he/she was traced on more than one occasion [21, 22]. These centers were located at the ALS Regional Expert Center, University of Turin, Piedmont, in the ALS Clinic in Bari, Puglia, the Motor Neuron Clinic at Beaumont Hospital, Dublin, Ireland, and the ALS Expert Center of Limoges teaching hospital.

### Statistical analysis

Patient baseline characteristics were reported as frequency (percentage) and mean with standard deviation for categorical and continuous variables, respectively. Survival analysis was performed using Kaplan-Meier analysis where day 0 was the date of diagnosis and the primary endpoint was tracheotomy-free survival for Italian and Irish cohorts (date of last follow-up of patients was July 2014) and overall survival for the French cohort (date of last follow-up of patients was November 2014).

Differences between each referral center and its corresponding registry were assessed using the Student’s T-test for continuous variables and the Pearson χ2 test for categorical variables.

In these comparisons, the assumption is that the means and the frequencies of the registries are the true values of the whole target population (when an expected frequency is zero, it is approximated to a number slightly different from zero to avoid computation problems).

A p-value of 0.05 was assumed to be the threshold for statistical significance.

Statistical analyses were performed using SPSS software (version 12.0.1, SPSS Inc, Chicago, IL), SAS v9.3 (SAS Institute, Cary, NC), R (v 3.3.1) and Rstudio (v 1.0.153). We complied with the Strobe Guidelines [23].

### Ethical considerations

The study was approved by Ethics Committee of “Policlinico Bari” and each site had an independent approval of the population-based registry of the region or nation (for Ireland).

## Results

During the four-year study period between January 1, 1996 and December 31, 1999, 584 residents of Piedmont and 335 Irish residents were diagnosed as having suspected, possible, probable, or definite ALS according to EEDC. There were 130 ALS cases diagnosed in the population of Puglia during the two-year period January 1, 1998 to December 31, 1999. Finally, there were 322 cases diagnosed as having possible, probable, probable laboratory supported or definite EEDC-revised ALS during the fourteen-years period January 1, 2000 to July 31, 2013 in Limousin. These four groups represent the population-based cohorts used in this study.

Of these, 241 patients from Piedmont (41.3%) routinely attended the multidisciplinary tertiary referral center at the University of Turin, 84 patients from Puglia (64.6%) attended the tertiary referral center at the University of Bari, 222 Irish residents (representing 66.3% of the Irish ALS population) attended the ALS clinic in Dublin and 273 patients from Limousin (84.8%) attended the tertiary referral center from the teaching hospital of Limoges. These four groups represent the tertiary referral center cohorts used in this study.

Demographics and clinical features of the population-based cohorts and the corresponding tertiary referral-based cohorts are shown in Table 2.

**Table 2:** Demographics and clinical features of the Irish, Italian and French ALS population cohorts and the corresponding tertiary referral cohorts.

		Piedmont (Italy)			Puglia (Italy)			Republic of Ireland			Limousin (France)		
		PARALS Register	Turin ALS		SLAP	Bari ALS		Irish ALS	Dublin ALS		FRALim	Limoges ALS	
		Register	Referral center		Register	Referral center		Register	Referral center		Register	Referral center	
		(n = 584)	(n = 241; 41.3%)	p value	(n = 130)	(n = 84; 64.6%)	p value	(n = 335)	(n = 222; 66.3%)	p value	(n = 322)	(n = 273; 84.8%)	p value
Mean age at diagnosis (SD)		65.3	62.9 (11.7)	0.001	62.3	60.7 (12.4)	0.240	64.0	63.2 (12.6)	0.345	68.7	67.8 (11.3)	0.189
Male cases		322 (55.1%)	133 (55.1%)	0.987	81 (62.3%)	48 (57.1%)	0.329	182 (54,3%)	122 (54,9%)	0.851	182 (56,5%)	154 (56,4%)	0.970
Familial ALS cases		11 (1.9%)	7 (2.9%)	0.243	2 (1.5%)	1 (1.2%)	0.795	11 (3.3%)	8 (3.6%)	0.789	22 (6.8%)	21 (7.7%)	0.573
Site of symptom onset:*				0.812			0.996			0.552			0.803
	Bulbar	216 (37.0%)	82 (34.0%)		34 (26.2%)	21 (25.0%)		125 (38.5%)	77 (35.3%)		99 (30.9%)	87 (32.0%)	
	Limb	364 (62.3%)	157 (65.1%)		96 (73.8%)	63 (75.0%)		171 (52.6%)	125 (57.3%)		221 (69.1%)	185 (68.0%)	
	Respiratory	4 (0.7%)	2 (0.9%)		0	0		0	0		0	0	
	Generalized	0	0		0	0		29 (8.9%)	16 (7.3%)		0	0	
Mean diagnosis delay (SD)¶		10.5	10.3 (9.0)	0.730	14.2	14.6 (14.0)	0.794	9.3	9.0 (11.3)	0.741	11.9	11.9 (12.4)	1
Median survival from onset (months)†		28.9	33.3	-	27.2	25.6	-						
Median survival from diagnosis (months)†								16.0	18.3	-	15.6	17.3	-

* Data was missing for 10 patients in the Irish ALS population (5 patients attending Dublin ALS referral center), 2 patients in the Limousin ALS population (1 patient attending the Limoges ALS referral center)

¶ data was missing for 119 patients in the Irish ALS population (67 patients attending Dublin ALS referral centre)

† Data was missing for 3 patients attending the Limoges ALS referral center

There were no statistically significant differences between the overall ALS population-based cohorts and the corresponding ALS tertiary center cohorts, except for the Piedmont.

However, patients attending a tertiary referral center were younger than the corresponding population cohort. This difference in average age at diagnosis was most marked in Piedmont, where patients attending the ALS referral center were an average of 2.4 years younger than the overall Piedmont ALS population (p = .001). The corresponding differences were 1.6 years for Puglia (p = .240), 1.1 year for Limousin (p = .189), and 0.8 years for Ireland (p = .345) but not significant. Male percentages appeared similar between ALS population and ALS referral cohort in Ireland, Piedmont and Limousin. As for Puglia, despite the non-significance (p = .329) there was a greater difference in the male percentage between the ALS population (62,3%) and the referral-cohort (57,1%).

The rate of fALS was reportedly higher in Irish and French as compared to the Italian cohorts. Patients with familial disease were more likely to attend the ALS centers in Dublin, Turin and Limoges.

No significant differences in site of symptom onset between ALS population and referral-cohort were found.

Nevertheless, it is possible to notice that with the exception of the French cohort, patients with bulbar-onset ALS were overall underrepresented in referral centers. In the Irish ALS referral center, 35.3% of patients described dysphagia or dysarthria as their initial symptoms compared 38.5% in the Irish ALS population. The same trend was observed in Italy.

While diagnostic delay appeared different among countries (e.g. 9.3 months in Ireland, 14.2 months in Puglia, 11.9 months in Limousin and 10.5 months in Piedmont) there was no significant difference between overall ALS populations and ALS referral cohorts.

Finally, patients attending the referral centers tended to live longer than the general ALS populations even if not significant. As compared to the median survival for the ALS population-based cohort, the increases for the corresponding referral cohorts were of 11% in Limousin, 15% in Piedmont and 14% in Ireland. There was no relevant difference in Puglia.

## Discussion

### Main results

In this paper, we analyzed data from four large population-based epidemiological ALS studies and determined that the clinical features and survival patterns of patients attending ALS referral centers tended to differ from the general ALS population. Patients referred to ALS centers tended to be younger (ranging from 0.8 to 2.4 years on average), less likely to present a bulbar onset, had a higher proportion of familial antecedents and a longer survival (ranging from 11% to 15%) as compared to the underlying ALS population. This study suggests a possible trend to referral bias in cohorts of ALS patients drawn solely from referral or hospital databases. We are obviously aware of the fact that it is difficult to draw conclusions on the general referral bias extent because of the between-population variability. The sources of this phenotypic variability are several and often unknown but one is surely genotype [19, 24].

### Difference between centers

The proportion of ALS patients attending referral centers varied from 41.3% in Piedmont to 85.0% in France, indicating indirectly the proportion of patients referred to primary and secondary care is still relatively large in some regions of Italy.

A number of interdependent factors influence the referral pattern to a particular ALS center including (i) organization of ALS care at the national level, (ii) local healthcare infrastructure, willingness to refer to a more specialized care among local neurologists and physicians, geographical location, (iii) sociocultural and socioeconomic level of the patients, and (iv) physical capacity for patients to go to ALS referral center. In this case, the distance to the referral center becomes relevant even in the early phase of the disease especially for more severe or older cases.

Overall, these factors determine the proportion of the general ALS population receiving their care at the specialist ALS clinic. At the time of data collection, almost two-thirds of all ALS patients in Ireland and Puglia and five-sixths in Limousin attended the regional ALS center and consequently the clinical features of these referral cohorts were the most similar to the corresponding ALS populations. In contrast, less than half of ALS patients recruited in the population-based registry in Piemonte, attended the Turin ALS center. Hence, our data suggests that it is not possible to estimate the extent of a referral bias in a clinic without first performing a direct comparison with the ALS population within the geographic area of interest.

### Selection bias and time

The proportion of patients with ALS in the population who were referred to the ALS centers tended to increase with time in our study. This reflected in an increasing similarity between referral and population-based samples. However, although one may expect that in the future, patients seen in ALS centers will be a representative sample of the underlying ALS population, the apparent reduction of selection bias in our study may be related to the fact that referral centers and population registries in the setting that were explored in this study are run by the same investigators. For this reason, we expect that in geographic areas with no population registries the differences between referral patients and the underlying ALS population are probably greater. It is possible that an ALS registry increase the awareness of ALS and of possible best pathways for cure in the population of that geographic area. The proportion over time of subjects shifting from primary and secondary care to tertiary ALS care in the area of a new population-bases registry is the measure of reduction of the possible bias and at the same time of the improvement of ALS care.

### Ageing issue

We showed here that younger patients have a more likely access to ALS referral centers. This is probably related to the fact that young patients are more likely to seek highly specialized care or to be referred to them by caring physicians [25]. Younger cohorts from ALS referral centers might drive at least partially the differences between the overall ALS population and the ALS referral cohorts in terms of clinical characteristics (i.e. proportion of bulbar onset cases) and prognosis (i.e. survival) [26].

Western populations are becoming older, leading to an increase in mean age at time of ALS diagnosis. For example, in our study mean age was comprised between 64.0 to 65.3 years for the Irish and Italian cohorts (created before 2000), while it was 68.7 years in the French cohort (created after 2000). We can then expect, in each site, a possible progressive rise of referral bias, in relation with the ageing of the population (and independently from the exhaustiveness pattern of the ALS clinic) [27]. The proportion of older people increases the number of subjects who do not reach the multidisciplinary clinic. This is due to the characteristics of rapid serious impairment in ALS elderly and to the trend to stay in facilities near home for elderly care.

### fALS

The definition of what is a familial case is difficult and inconsistent across neurologists, including ALS experts [28]. In a referral center, ALS specialists may be more aware of familial ALS and the importance of different genotypes and therefore more keen in the identification of familial cases. Population-based epidemiological studies have shown the rate of fALS to be between 1.5% and 6.5% [10, 11, 13, 14, 29], with a pooled proportion estimated at 4.7% by a meta-analysis [28], whereas referral-based studies consistently reported rates of about 10% [30, 31]. A detailed case control study with verification by death certification, and associated interrogation of data held in a population-based register has suggested that the rate is up to 15% In Ireland [32].

Piedmont patients with a family history of ALS were nearly 60% more likely to be referred to an ALS clinic. Patients with a family history are often more knowledgeable about the disease and the healthcare resources available within their community. This patient group is also motivated to seek out expert opinion concerning the genetic risk to other family members. The phenotype of familial ALS patients may differ from that of sporadic patients in relation with the underlying high-risk gene implicated [33]. fALS patients tend to be younger, have slower progression rates and equal male and female incidence [34]. Though the absolute numbers are low, a higher proportion of familial cases may influence the overall clinical characteristics of a clinical-based cohort. Familial aggregation precise estimates are important both for genotype prevalence and environmental exposure, especially early in life [32].

### Bulbar onset

The proportion of bulbar-onset ALS patients varied considerably from one country to another, from 38.5% in the Irish ALS cohort and 37% in Piedmont, vs. 31% in Limousin and 26% in Apulia, as previously described by the EURALS Consortium [7]. Except for Limousin, referral patients had lower proportions of bulbar-onset cases than patients from population registries. The difference in in prevalence of bulbar phenotype across registries and clinical center may determine the difference in prognosis across the two type of settings. The bulbar has a considerable worse prognosis according to most studies (up to 16 months shorter than limb-onset disease [35]). The site of onset and age may have profound impact on the evaluation of prognosis. This has be taken into account when selecting patients for clinical trials in the process of evaluation of new drugs in tertiary referral center. It is still not clear if this difference across European countries is real or due to different clinical approach.

### Survival

Survival varied when ALS populations were contrasted to referral cohorts.

Other studies have shown that the difference in age and type of onset are responsible of these differences [26]. Selection of the samples due to the severity of the disease at time of diagnosis, tends to exclude from referral cohorts patients with unfavorable prognostic factors and therefore shorter survival. This characteristic may hamper the validity of the data from referral-cohorts in particular on prognosis.

### Previous studies in the field

Previous studies attempted to describe bias related to referral centers [36, 37]. They were criticized for some design characteristics: small study size, differences in term of geographical coverage between population-based and referral-based cohorts [36] or by the fact that they compared local prevalent cases versus the populations attending a referral center [37], which might have skewed their results [38].

Lee et al. [36] identified referred patients to be younger (57.3 vs 59.9 years), more frequently male (63% vs 52%), with a higher proportion of fALS cases (5.0% vs 2.1%), a higher proportion of upper extremity onset and a longer diagnostic delay (12.0 months vs 9.0 months). Interestingly, they also displayed different referral patterns depending on ethnicity (5% of blacks in the referral cohort as compared to 16% in the incident cohort), reflecting difficulties to access of care for some population groups. Effects of unfavorable prognostic factors were stronger in the incident cohort and characteristics of referral patients were more homogeneous. While within the first three years survival was not dramatically different between cohorts, it was 21% in referral cohort and 4% in incident cohort at five years. Sorenson ed al [37], based on Mayo clinic ALS database, found a 11-month difference in survival between local patients (median survival time 18 months) and referred patients (median survival time: 29 months, p = 0.007). The referred patients had a 5-month delay in the first appointment to the ALS clinic, more riluzole and non-noninvasive positive pressure ventilation prescription. No difference was identified in terms of age, gender or bulbar onset.

The above-mentioned differences have also been identified by the few studies that attempted to assess the efficacy of multidisciplinary care in ALS [21, 22, 39]. Comparing patients from general neurology clinics and patients underlying multidisciplinary care (MDC), Traynor et al. [21] found MDC patients to be younger by 5.5 years, more frequently self-reporting as fALS cases (12.2% vs 2.7%), and bulbar-onset patients (40.8% vs 34.1%). Chiò et al. documented a younger age for referred patients (4.2 years), but did not identify differences in terms of sex, type of onset, and diagnostic delay [23]. In both studies, referred patients, who also had a greater access to riluzole and combination of all available strategies (increased use of non-invasive ventilation, attention to nutrition and earlier referral to palliative referral services [8]), displayed longer median survival times, of respectively 7.5 months and 10.0 months. However, while the populations were different, multivariate analysis revealed an independent effect on improvement of the survival of the multidisciplinary care, a finding which was recently replicated [40, 41]. Notwithstanding, the evidence for its effectiveness is considered as unclear (“low and very low quality of evidence”) by the Cochrane group [42] who considers the benefit for survival as “conflicting”.

### Consequences for ALS research

This study provides possible evidence that patients form tertiary centers are self-selected and may be not entirely representative of the reference ALS population. Remarkably for clinicians, the phenotype of ALS at beginning of disease is different in some important traits between populations [41]. Hence, the studies from clinical cohorts focusing on phenotype, prognosis or biomarkers are likely to provide point estimates that are influenced by the setting. As recruitment of ALS patients in RCT is generally based on ALS tertiary centers, this might also significantly affect the results of experimental studies. While this issue is up to now largely undervalued [43], we can postulate that groups of ALS patients included in RCT are younger, less likely women, with longer diagnostic delay, more frequent spinal onset and fALS [44]. This selection, favoring the subjects with better prognosis, could at least partially help to explain the negative results of ALS trials.

Our study showed some differences when contrasting patients from different registries. Some difference may be real but could be also due to subtle differences both in populations and in practice across different centres in Europe. Variation of ALS clinical presentation and survival between Northern, Western and Southern European sub-continents was recently explored and discussed [41].

The reported differences cannot be explained solely by the small differences in age at onset and other known and unknown factors may play a role. This finding, implies the need to consider the country of origin, a possible surrogate of the underlying genotype, as an important possible modifier of the effects of any experimental treatment.

An additional limitation of our study is the lack of data on percentage of diagnoses not confirmed by neurologists in the tertiary centers or in the registry. The percentage of change in ALS diagnosis is 7% after three years in European registries [45]. For further studies it would be interesting to analyse the degree of agreement between diagnosis in different care settings.

In conclusions the extent and possible causal components of recruitment in clinical series from referral centers need to be carefully examined and contrasted possibly with the results of population-based studies in the same geographic area. The presence of a registry especially if stable over several years [46] may enhance the referral of ALS patients to tertiary center of the area over time. The ongoing population-based registries in Europe may add useful information on results and methods of clinical ALS studies enhancing the quality of this process in tertiary referral centers [8, 46].

## Supporting information

S1 File Databases. (ZIP)
